# Endoscopic management of colorectal polyps

**DOI:** 10.1093/gastro/goad027

**Published:** 2023-05-27

**Authors:** Pingting Gao, Kaiqian Zhou, Wei Su, Jia Yu, Pinghong Zhou

**Affiliations:** Endoscopy Center and Endoscopy Research Institute, Zhongshan Hospital, Fudan University, Shanghai, P. R. China; Surgery Department, Zhongshan Hospital, Fudan University, Shanghai, P. R. China; Endoscopy Center and Endoscopy Research Institute, Zhongshan Hospital, Fudan University, Shanghai, P. R. China; Surgery Department, Zhongshan Hospital, Fudan University, Shanghai, P. R. China; Shanghai Medical College, Fudan University, Shanghai, P. R. China; Endoscopy Center and Endoscopy Research Institute, Zhongshan Hospital, Fudan University, Shanghai, P. R. China

**Keywords:** therapeutic endoscopy, polypectomy, colorectal polyps, colonic lesion

## Abstract

Colorectal polyps are premalignant lesions in the lower gastrointestinal tract. Endoscopic polypectomy is an effective strategy to prevent colorectal cancer morbidity and more invasive procedures. Techniques for the endoscopic resection of polyps keep evolving, and endoscopists are required to perform the most appropriate technique for each polyp. In this review, we outline the evaluation and classification of polyps, update the recommendations for optimal treatment, describe the polypectomy procedures and their strengths/weaknesses, and discuss the promising innovative methods or concepts.

## Introduction

Colorectal cancer (CRC) is the third most diagnosed malignancy and second deadliest cancer in the world [1], causing almost 900,000 deaths annually [2]. The lifetime risk of developing CRC is ≥4.0% [3]. The carcinogenesis process of CRC includes four pathways: adenoma–carcinoma pathway, serrated neoplastic pathway, inflammatory pathway, and de novo pathway [1, 4]. The first two pathways account for the vast majority and arise from colorectal polyps. The conventional adenoma–carcinoma pathway leads to ∼70% of sporadic CRC [5] and the serrated neoplastic pathway accounts for 15%–30% of CRC [6, 7]. Colorectal polyps are the precursors of CRC and precede long before carcinogenesis, which presents opportunities to prevent cancer by removing precursor lesions. Nowadays, screening and resection of colorectal polyps by endoscopy is the cornerstone in reducing the incidence and mortality of CRC [8–10].

## Assessment of colorectal polyps

Colorectal polyps are defined as discrete abnormal tissue masses protruding into the lumen of the colon or rectum [11]. These polyps are attached to the mucous membranes of the lumen wall either by stalk or broad base [11, 12]. The detection of colorectal polyps always comes with the questions of whether they are benign or malignant and whether they will always stay benign or become malignant one day. Such process of diagnosis is also called “optical biopsy” [13]. More often, endoscopists are required to evaluate the biological behavior of the lesion in order to make a treatment strategy. Therefore, careful observation of colorectal polyps should be first carried out under endoscopy.

Colorectal polyps have a variety of features. Their macroscopic characterization provides information to predict the histology, biological behavior, and prognosis of the lesions. In that case, the endoscopic description of colorectal polyps should include location, size, morphology, suspected histopathology, and estimation of depth of invasion. The location can be described as proximal colon, distal colon, rectosigmoid, and difficult sites (such as the ileocecal valve, appendiceal orifice, and anorectal junction) [14]. The size can be stratified as diminutive (≤5 mm), small (6–9 mm), intermediate (10–19 mm), and large (≥20 mm) [15]. Morphologically, colorectal polyps can follow the Paris classification of superficial neoplastic lesions [16]: protruding types (I) contain pedunculated (Ip) and sessile (Is) lesions; non-protruding and non-excavated types (II) include slightly elevated (IIa), completely flat (IIb), and slightly depressed (IIc) lesions; an ulcer is seen in excavated type (III). Lesions assigned to Is/IIa/IIb/IIc with a size of >10 mm and extending laterally (in contrast to vertically) along the colonic wall are also known as laterally spreading lesions (LSLs) [17]. When colorectal polyps are closely observed using high-resolution narrow band imaging (NBI) endoscopy, the characteristics of (i) lesion color, (ii) microvascular architecture, and (iii) surface pattern (also called pit pattern) can be recognized [18]. Based on the three characteristics, NBI international colorectal endoscopic classification can be made and help to discriminate hyperplastic polyps (Type 1), adenomas (Type 2), and deep submucosa-invasive cancer (Type 3) [19]. Pathologically, colorectal polyps can be classified as adenomatous polyps and serrated polyps. Adenomatous polyps contain three histologically types: tubular adenoma, tubulovillous adenoma, and villous adenoma [11, 20]. Serrated polyps are a heterogeneous group of lesions that could be further classified into three categories: hyperplastic polyps, sessile serrated lesions, and the traditional serrated adenomas (TSAs) [21]. The methods for assessing the depth of invasion into the submucosal layer include the Haggitt classification [22] for pedunculated lesions and the Kikuchi classification [23] for non-polypoid lesions. Following the detection and careful evaluation, endoscopists can subsequently choose the optimum treatment for colorectal polyps.

## Current status of polypectomy-related publications

Qualitative and quantitative analyses of relevant studies from the past 5 years were performed using Bibliometrix to provide a comprehensive insight into the research context of polypectomy. Data from 2018 to 2022 were retrieved from the Web of Science core collection in November 2022. Scientometric analysis of the data was performed using the Bibliometrix R package (4.2.2) and the VOS viewer (1.6.18) in this study.

**Figure 1.**
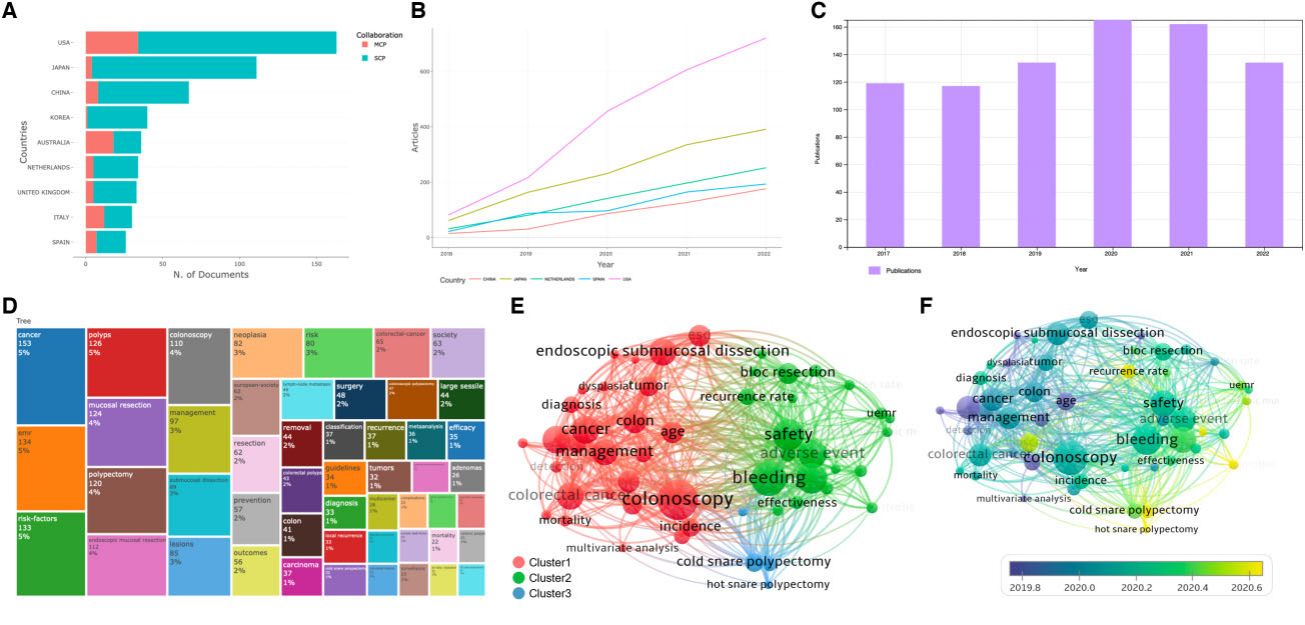
Current status of polypectomy-related publications. (A) and (B) The top five countries in terms of number of publications; (C) publications in the past 6 years; (D)–(F) keywords related to polypectomy were analysed and demonstrated. MCP, multiple country publications; SCP, single country publications.

A total of 679 articles were retrieved with a total of 4,984 citations, an average of 7.34 citations per item, and an H index of 34. The research articles (564 of 679) constituted the bulk of the published items and the rest were reviews (115 of 679). In total, 39 countries contributed to these research publications. In terms of the number of publications, the top five countries were the USA (*n *=* *163), Japan (*n *=* *111), China (*n *=* *67), Korea (*n *=* *40), and Australia (*n *=* *36) in sequence (Figure 1A). Additionally, all these five countries have witnessed a steady increase in publications in the past 5 years (Figure 1B). Although the total number of papers published on polypectomy topics underwent a slight decline in 2021 and 2022, the annual production remained high from 2018 to 2022 (Figure 1C), indicating a robust and enduring interest in polypectomy. Figure 1D–F demonstrates keywords related to polypectomy analysed by the VOS viewer. Figure 1D shows the overall picture of keywords related to polypectomy and the area on the chart is proportional to the number of occurrences of each keyword. As the most widely studied polypectomy procedure in the past 5 years, the keyword “EMR” takes up the largest portion of the chart (5%). With increasing attention on risk factors of various polypectomy procedures, the keyword “risk factors” also accounts for the maximal portion (5%). All of the keywords were classified into three clusters (Figure 1E). Cluster 1 consists of 27 keywords, mainly about the exploration of the clinical application of polypectomy; Cluster 2 consists of 18 keywords, focusing on the post-operative complications of polypectomy; Cluster 3, which consists of three keywords, sheds light on the heated discussion of cold vs hot snare polypectomy lately. Studies up to 2020 focused mainly on the exploration of the scope of application, whereas studies beyond 2020 showed more interest in the evaluation of outcomes after polypectomy and corresponding treatment (Figure 1F). In a nutshell, these results indicate a strong and growing interest in polypectomy, which holds a promising future as well.

## Recommended resection techniques for colorectal polyps

**Figure 2.**
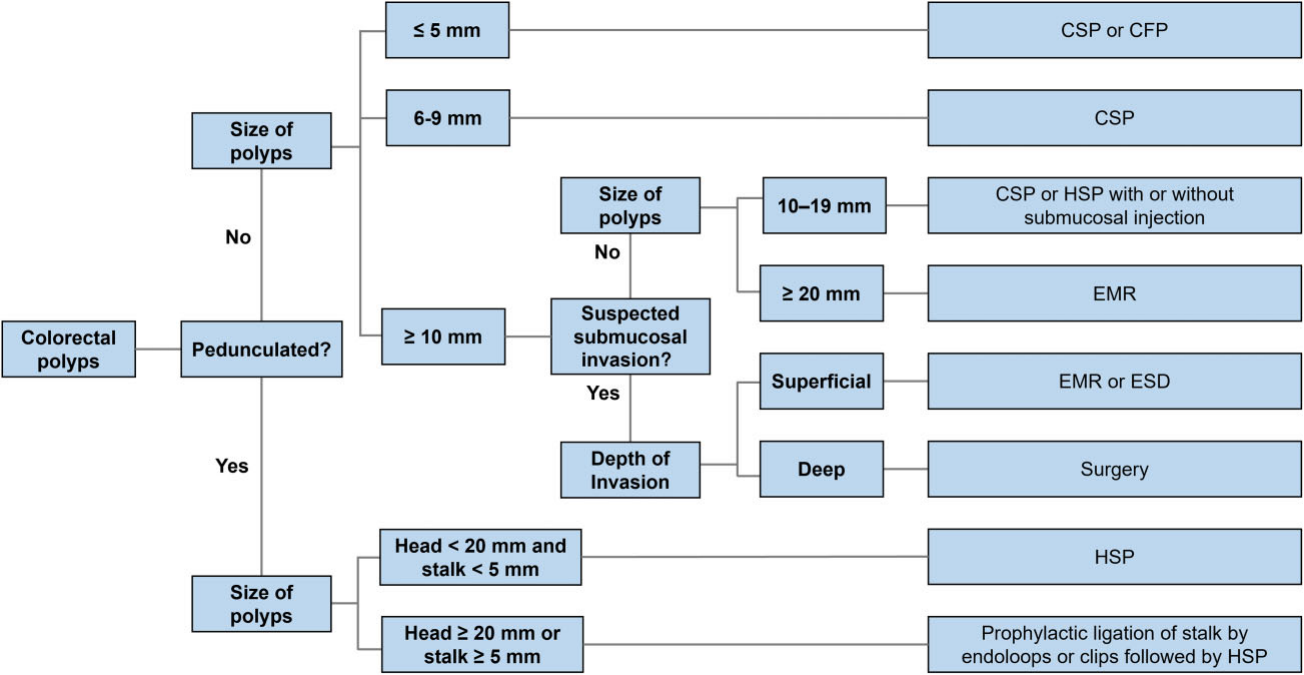
Recommended resection techniques for colorectal polyps. CSP, cold snare polypectomy; CFP, cold forceps polypectomy; HSP, hot snare polypectomy; EMR, endoscopic mucosal resection; ESD, endoscopic submucosal dissection.

Complete resection of colorectal polyps and prevention of CRC are the aims of endoscopic polypectomy. Therefore, the endoscopist should choose the most complete, safest, and most effective and evidence-based resection technique. An algorithm of polypectomy recommendations is as follows [15, 24–26] (Figure 2) :

- For pedunculated colorectal polyps with a head of <20 mm and a stalk of <5 mm in diameter, hot snare polypectomy (HSP) is recommended.
- For pedunculated colorectal polyps with a head of ≥20 mm or a stalk of ≥5 mm in diameter, the risk of post-polypectomy bleeding increases because of the large vessel within the stalk. Thus, prophylactic ligation of stalk by using endoloops or clips is recommended prior to HSP.
- For non-pedunculated diminutive (≤5 mm) and small (6–9 mm) lesions, it is recommended to use cold snare polypectomy (CSP) to achieve en bloc resection. If CSP is technically difficult (lesion of ≤3 mm, at difficult sites), jumbo or large-capacity cold biopsy forceps polypectomy may be considered.
- For non-pedunculated lesions of ≥10 mm, advanced endoscopic imaging is recommended to identify the presence of submucosal invasion such as irregular or absent surface vascular patterns.
- For non-pedunculated non-invasive lesions with intermediate size (10–19 mm), CSP or HSP with or without submucosal injection is recommended.
- For non-pedunculated non-invasive lesions with large size (≥20 mm), endoscopic mucosal resection (EMR) is recommended. All grossly visible tissue should be resected in one session, and the post-EMR margin without visible remains should receive adjuvant thermal ablation.
- For non-pedunculated lesions suspected of superficial submucosal invasion, EMR or endoscopic submucosal dissection (ESD) is recommended if complete resection is feasible and safe.
- For non-pedunculated lesions suspected of deep submucosal invasion, surgery is recommended.

## Endoscopic resection techniques

### Biopsy forceps polypectomy

**Figure 3.**
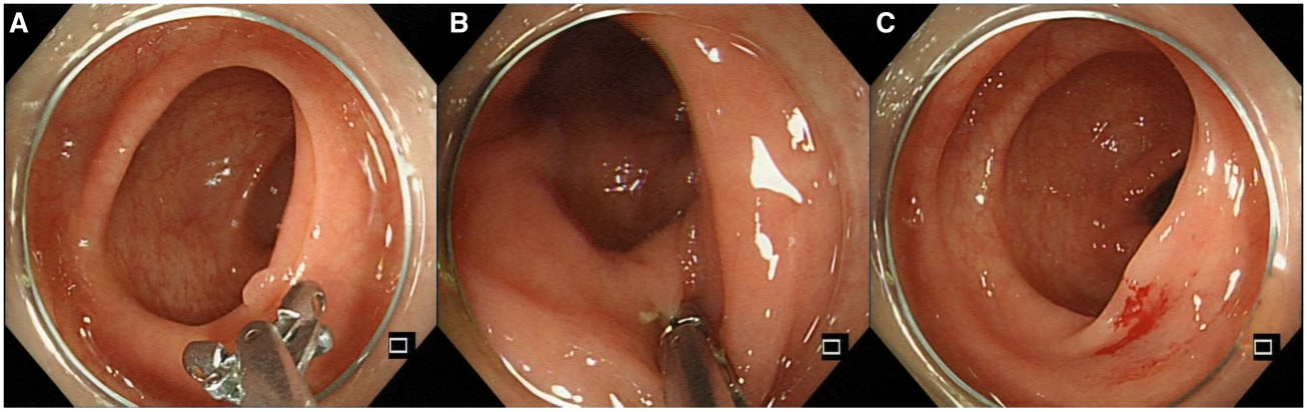
Biopsy forceps polypectomy (BFP) procedure. (A) The colorectal polyp and biopsy forceps; (B) the action of biting off; (C) the scene after BFP.

Biopsy forceps polypectomy (BFP) bites the polypoid tissue off by using the biopsy forceps. Then, the specimen is retrieved directly by using the biopsy forceps (Figure 3). Additional biopsies are taken if residual polypoid tissue is suspected. Biopsy forceps can be classified as hot biopsy forceps or cold biopsy forceps, depending on whether to use electrocautery or not. Cold forceps polypectomy (CFP) carries advantages such as the widespread and immediate device availability and simplicity of use. However, the incomplete resection rates of CFP rise along with the lesion size, the number of bites, and the obscure bleeding field after the initial bite [27]. Hot forceps polypectomy (HFP) can be applied to control bleeding [28]. However, it is recommended against the use of HFP for polypectomy because of the high rates of incomplete resection, inadequate tissue sampling for histopathological examination, and high risks of adverse events in comparison with snare excision [15]. According to the jaw volume, biopsy forceps can also be classified as large-capacity forceps and jumbo forceps [29]. The latter have a larger cup. Jumbo forceps are superior to the standard forceps in achieving complete visual endoscopic resection of sessile polyps measuring ≤6 mm and reducing the procedure time [29]. In practice, BFP is the most used polypectomy technique for polyps of ≤3 mm in that it is simple to use and there is no significant difference with CSP in complete resection rates or adverse events [27]. When a diminutive polyp is in a position that is difficult to snare, BFP, especially using jumbo forceps, is also an alternative and applicable technique [30].

### CSP

**Figure 4.**
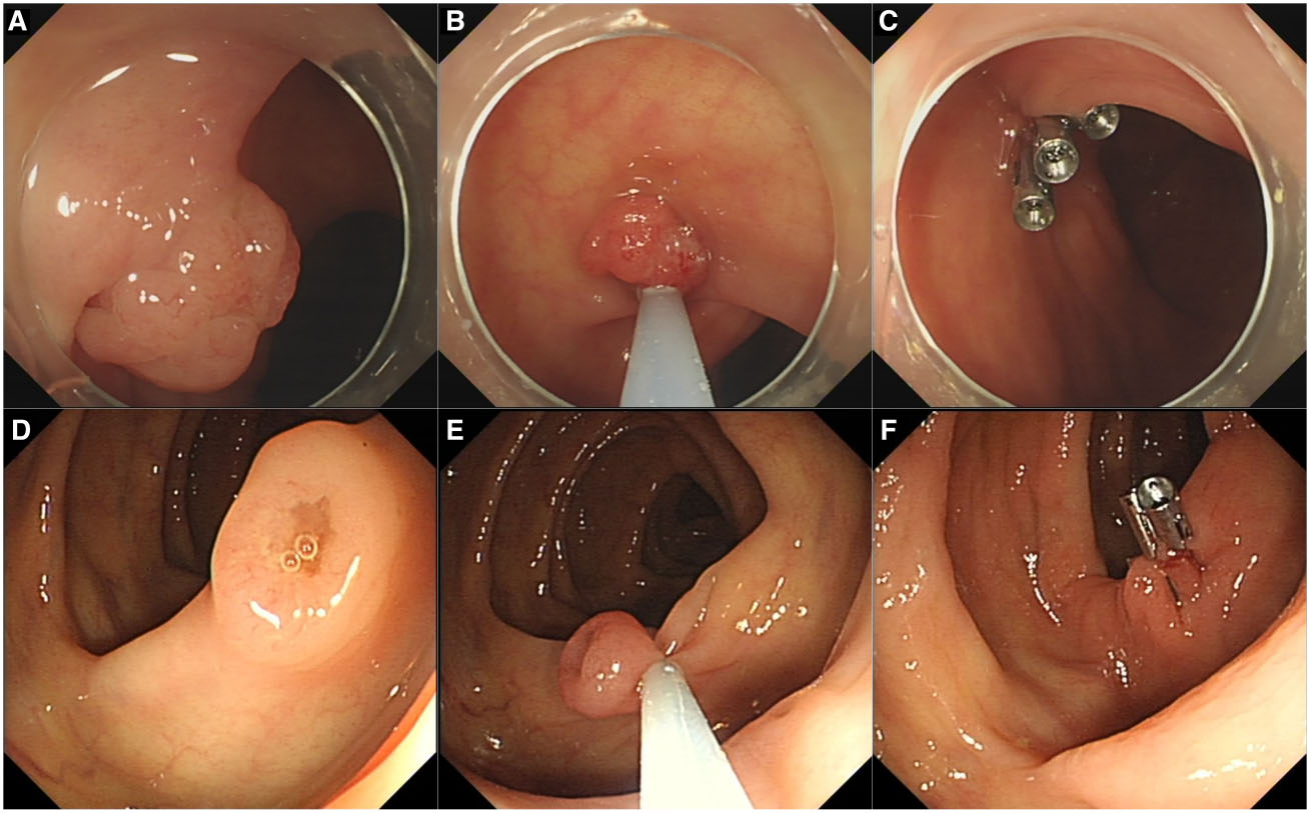
Cold/hot snare polypectomy (CSP/HSP) procedure. (A)–(C) CSP procedure; (D)–(F) HSP procedure (with electrocautery).

CSP is a simple resection method for completely resecting small polyps. In detail, the polyp with a margin of 1–2 mm of normal tissue around the base is encircled, grasped, and resected by using a snare without using electrocautery. Then, the specimen is suctioned through the colonoscope channel into a trap (Figure 4A–C). Its advantages in shortening procedure time and lowering treatment costs also contribute to the wide use of CSP in practice [31]. For polyps of <5 mm, CSP has a higher rate of complete resection than HFP [32] and CFP [33]. For colorectal polyps sized ≤10 mm, although CSP and HSP are the same in efficacy, CSP is safer than HSP. A meta-analysis integrating 32 randomized–controlled trials (RCTs) showed that the pooled incomplete resection rate of CSP and HSP is no different [34]. In addition, a large-scale RCT reported that the delayed bleeding rate was significantly lower in CSP than in HSP [35]. Therefore, the international guidelines recommend CSP as the first-line treatment for small colorectal polyps (measuring <10 mm) [15, 25]. When performing CSP, it is also recommended to use a thin wire snare specifically designed for CSP and to observe the surrounding mucosa of the resection site using chromoendoscopy or image-enhanced endoscopy to ensure that there is no residual lesion [31].

### HSP

HSP is defined as the snare resection for polyps involving electrocautery. The procedure is as follows [36]. First, a snare is opened and encircles the polyp. Then, the snare is closed slowly and progressively, capturing 1–2 mm of normal tissue around the polyp. Next, the electric current and mechanical traction are applied to the polyp by using the snare. Finally, the polyp is cut (Figure 4D–F). HSP differs from CSP mainly in the use of electrocautery, which brings advantages along with disadvantages. The ability to cut and coagulate simultaneously enables HSP to deal with small vessels supplying the polyps [37]. Although HSP could be used to remove pedunculated lesions, polyps with larger sizes require additional ligation of large vessels within the stalk [15]. On the other hand, electrocautery results in thermal damage to the deep layers of the colon wall, which may induce tissue necrosis that proceeds laterally [38]. HSP was not recommended for small non-pedunculated lesions, due to the higher risks of abdominal pain and delayed hemorrhage compared with CSP [39, 40]. The side effect of electrocautery may be alleviated by submucosal injection [41].

### Argon plasma coagulation

**Figure 5.**
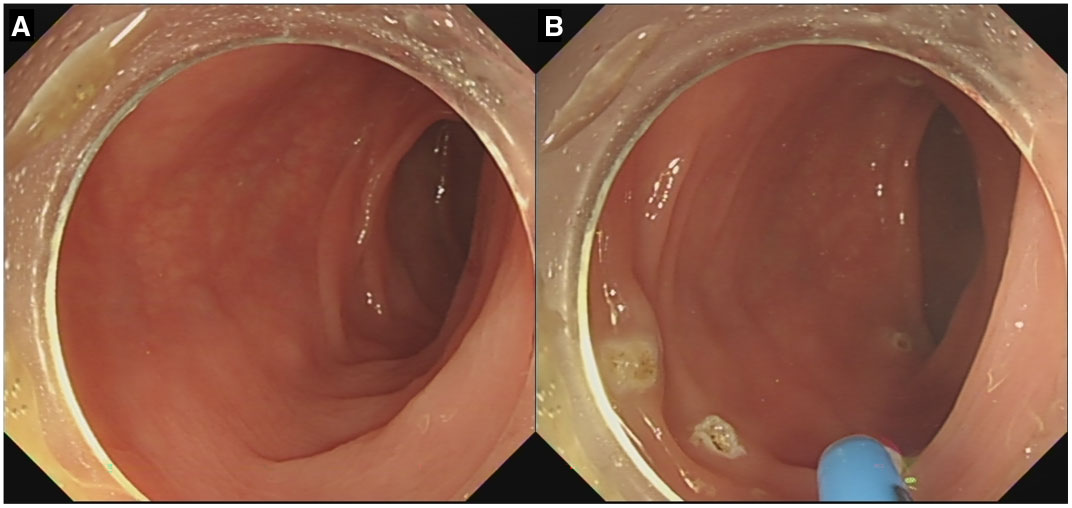
Argon plasma coagulation (APC) procedure. (A) The scene before APC; (B) the scene after APC.

Argon plasma coagulation (APC) is a noncontact ablation technique. When performing APC, ionized argon gas (known as gas plasma) is used to deliver the energy with a conductive property to ablate the target tissue in a non-touching way (Figure 5) [42, 43]. APC is easier, quicker, and safer compared with other thermal/laser therapy. Its disadvantages lie in the serious complications of perforation, gas explosion, and submucosal injection of argon gas through inadvertent contact [44]. Since its introduction to endoscopy, APC has been reported to manage hemostasis, angiodysplasia, radiation proctitis, and tumor debulking [44]. APC is also a promising adjuvant treatment for large non-pedunculated lesions. Applying APC after piecemeal snare polypectomy or EMR may help eradicate microscopic polyp remnants on the margins or at the base [43, 45–48]. Recently, two prospective studies demonstrated the efficacy and safety of adjuvant APC after EMR in treating non-pedunculated colorectal polyps of ≥20 mm by showing a low recurrence rate (0–2.2%) and low adverse event rate (4.8%–7.5%) [43, 48]. A large RCT is still needed to confirm the results. Moreover, when polyps recur at the site of prior polypectomy, the massive submucosal scarring makes additional resection challenging. In that case, the application of APC, preceded by submucosal injection to protect the muscle layer, would assist in the complete eradication of recurrent fibrotic colon polyps [49]. The thermal effect on tissue is influenced by the duration of application, power output, and distance between the probe and the target [42]. Although the thermal effect usually occurs to a limited depth, APC applied to the same area for a prolonged period can cause transmural injury and perforation [42].

### EMR

**Figure 6.**
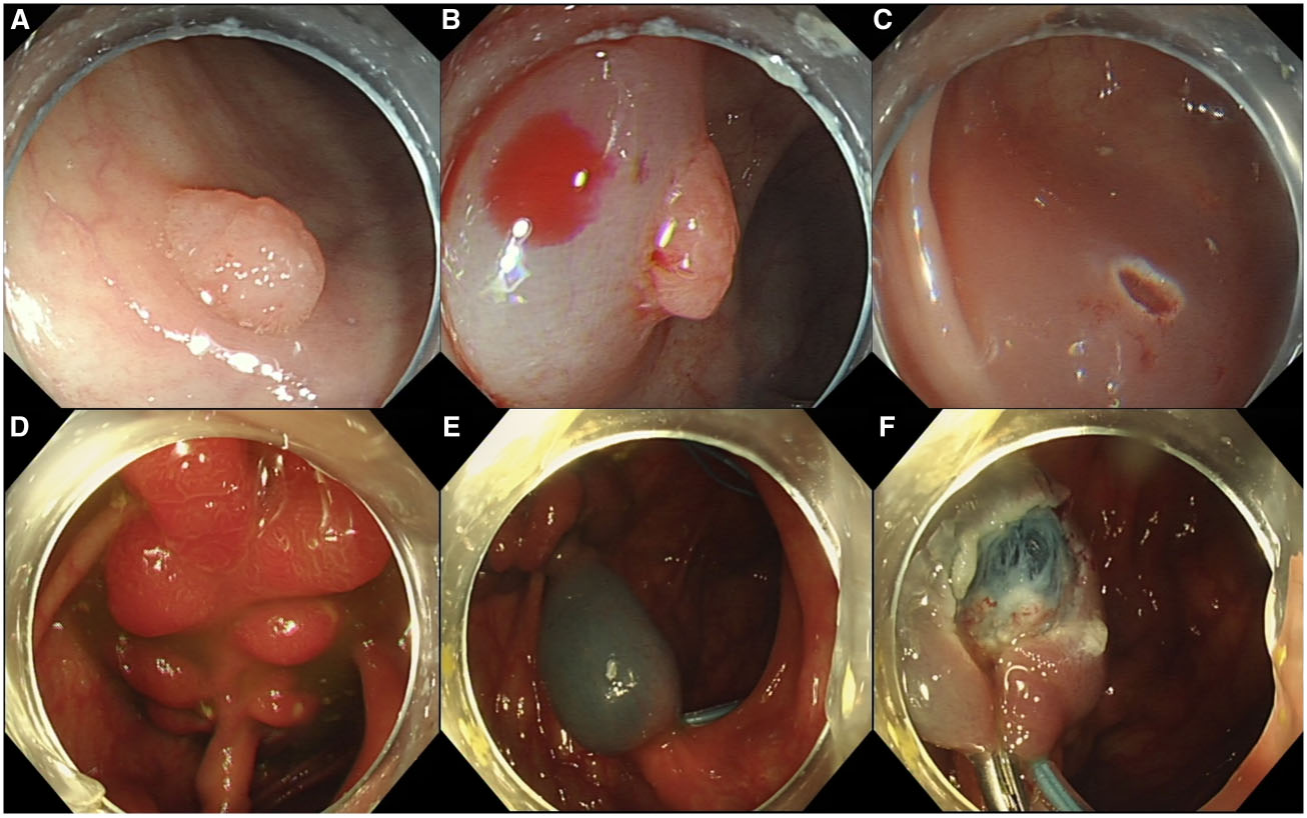
Endoscopic mucosal resection (EMR) procedure. (A)–(C) Small polyp (<2 cm) resected by using EMR; (D)–(F) polyp of >5 cm resected under EMR with the help of a nylon loop.

EMR involves injections of a solution (normal saline solution or viscous solution, e.g. sodium hyaluronate, succinylated gelatin, hydroxyethyl starch, and glycerol) into the submucosal space of a superficial lesion through an injection needle [25]. In our center, we prefer using a mixture of methylene blue and saline. The lesion is then resected using a snare by applying high-frequency current (Figure 6). Although growing evidence shows the benefit of fewer adverse effects in cold snare EMR (EMR without applying high-frequency current) [50], conventional hot snare EMR (EMR using high-frequency current) is still essentially applied [15]. Lesions of <20 mm usually can be removed in a single piece, whereas lesions of ≥20 mm are more likely require piecemeal resection, namely piecemeal EMR. In piecemeal EMR, a lesion is first cut into a large piece to accurately carry out histological diagnosis and the residual part is then deliberately cut into pieces [24]. Precutting EMR describes a technique wherein snaring is carried out after incising the circumference of the lesion by using the tip of a snare or a knife for ESD. Large prospective studies have demonstrated EMR as a safe, efficient, and cost-effective procedure [51]. EMR is a fundamental technique for endoscopic resection. Although EMR can be applied to large lesions, incomplete resection and poor outcome cannot be avoided [43, 52, 53]. Features associated with incomplete resection or recurrence include lesion size of >40 mm, ileocecal valve location, prior failed attempts at resection, and size, morphology, site, and access (SMSA) level 4. Careful lesion assessment prior to EMR is recommended [15]. For EMR, all efforts should be made to ensure the absence of neoplastic tissue at the post-EMR mucosal defect and margin.

### ESD

**Figure 7.**
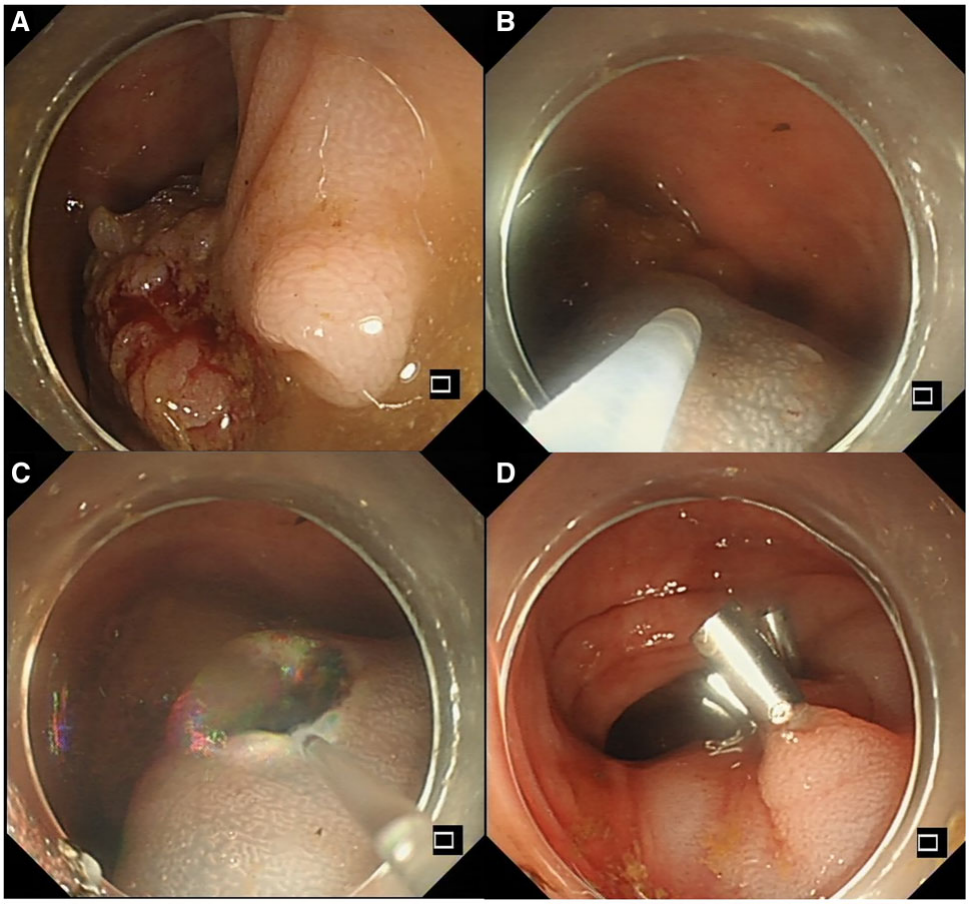
Endoscopic submucosal dissection (ESD) procedure. (A) A large thick pedunculated polyp under endoscopy; (B) injection; (C) incision and dissection; (D) closure of the wound.

ESD begins with marking the tissue margin of 3–5 mm outside the lesion. Subsequently, an injection procedure is performed that is similar to that in EMR. The fluid-expanded submucosal space separates the layers and offers precise control over resection depth and lateral extent. The circumference of the lesion is then incised using an endoscopic knife with an electrical cutting current and then the submucosa is dissected (Figure 7). This technique can resect the lesion in one piece regardless of its size. However, the technical complexity, risk of adverse events, and long procedure duration hamper the wide performance of ESD [54]. Simplified/hybrid ESD, which is characterized by partial submucosal dissection followed by snare-assisted resection, provides a bridge between conventional EMR and full ESD [55]. Since en bloc resection by using ESD has shown a lower rate of recurrence compared with piecemeal resection (1%–2% compared with 10%–20% by using ESD; odds ratio, 8.2), ESD offers high rates of curative resection [56]. What is more, the intact specimens produced by ESD allow more accurate pathological and oncological evaluation [57–59]. Both the European Society of Gastrointestinal Endoscopy (ESGE) and the Japanese Society of Gastroenterology (JSGE) recommend ESD for lesions that cannot be optimally and radically removed using snare-based EMR [56].

## Perspectives

Endoscopic therapy is simple, short in operation time, and minimally invasive, and is considered as the first-line treatment for colorectal polyps and early cancer [60]. It is very important to determine the nature of the lesions before treatment using ultrasound endoscopy and magnifying endoscopy to estimate the shape and depth of the lesions before operation. En bloc resection of the lesion is vital for pathological assessment and better prognosis [61]. In practice, the size, shape, depth of invasion, and individual risk factors should be fully considered in the selection of treatment methods to improve the feasibility, effectiveness, and safety of endoscopic resection.

Reflecting back updates in digestive disease week (DDW) 2022, more work has been done to help clinical decision-making. Combing precious work and experience at our center, we believe that the following research on endotherapy is promising. For example, submucosal epinephrine-added saline injection may reduce the time required for the CSP procedure and study has shown that cold snaring is a dominant resection technique for non-pedunculated colorectal lesions of 6–15 mm in size compared with hot snare resection [62]. The ongoing study of the safety and effectiveness of cold EMR for resection of large polyps of >2 cm compared with hot EMR is worth discussing and researching. For flat sessile lesions, precancerous lesions, and early cancers, EMR and ESD are more common. Many studies have been conducted to expand indications of different technologies, such as comparing cap-assisted EMR and ESD in non-lifting and adherent colorectal polyps. Suturing method developments have changed the way of thinking and perforation is no longer prohibited. For more advanced colorectal neoplasia, several established endoscopic methods exist from ESD to endoscopic full-thickness resection and a full-thickness resection device appears to be feasible and efficacious in the resection of benign neoplasms of ≤30 mm in diameter [63]. As we know, more work comparing ESD and endoscopic full-thickness resection in endoscopic therapy for colorectal neoplasia (size <3 cm) is in progress.

A lot of work has been done to reduce the recurrence rate. Previous study has concluded that large non-pedunculated colorectal polyps with covert submucosal invasive cancer following piecemeal EMR will have no residual malignancy and the risk of residual malignancy can be ascertained from three key variables: poor differentiation, lymphovascular invasion, and R1 deep margin [64, 65] and more meta-analyses have been started to find whether EMR/ESD along with routine margin ablation or snare tip soft coagulation could lower the recurrence rate; thus, these techniques should be considered as standard for endoscopic resection of large colorectal polyps, while CSP, cold EMR, and underwater EMR (UEMR) should only be used within clinical trials pending more high-quality data regarding the local recurrence rate. UEMR has emerged as an alternative method for conventional EMR as the standard modality for removing non-pedunculated colorectal lesions [66]; an RCT has been conducted to demonstrate a lower recurrence rate and shorter procedure duration by using UEMR.

More innovative combinations of medical science with engineering have emerged, such as the dual balloon endolumenal overtube platform to enhance scope stability and visualization, traction, and function as a conduit for scope exchange; a new ligation method using the double-loop clips technique without an adhesive agent for ulceration after ESD; a novel through-the-scope suture device (X-Tack, Apollo Endosurgery) consisting of four suture-connected tacks, allowing direct closure of large polypectomy defects; and a novel bipolar radiofrequency ablation knife approved by the Food and Drug Administration for the performance of ESD utilizing bipolar radiofrequency ablation current for submucosal dissection. At our center, we developed a novel plasma radiofrequency generator and its matched disposable electric snares for EMR.

Innovative methods and novel devices and suturing under endoscopy to reduce the difficulty of treatment and the incidence of complications are continually being updated and are potentially changing traditional ways of performing surgery.

## Conclusions

Endoscopic resection is the most important skill for treating colorectal polyps, gaining strong interest. The endoscopic assessment of polyps should identify characteristics of the location, size, morphology, suspected histopathology, and estimation of the depth of invasion. This review has summarized the procedures, features, advantages, side effects, and consensus-based recommendations of endoscopic practice for the resection of colorectal lesions. Endoscopic technology is still innovating and the results of studies on its effectiveness are to be expected.

## Authors’ Contributions

P.G. and K.Z. wrote the manuscript; W.S. collected the cases; J.Y. conducted the scientometric analysis of relevant research; P.Z. designed the article, provided the case, and made critical revisions. All authors have read and approved the final version of the manuscript.
